# Patient satisfaction of primary care for musculoskeletal diseases: A comparison between Neural Therapy and conventional medicine

**DOI:** 10.1186/1472-6882-8-33

**Published:** 2008-06-24

**Authors:** Joelle Mermod, Lorenz Fischer, Lukas Staub, André Busato

**Affiliations:** 1 Institute for Evaluative Research in Orthopaedic Surgery, University of Bern, Stauffacherstrasse 78, CH-3014 Bern, Switzerland; 2 Kollegiale Instanz für Komplementärmedizin (KIKOM), University of Bern, Imhoof-Pavillon, Inselspital, CH-3010 Bern, Switzerland

## Abstract

**Background:**

The main objective of this study was to assess and compare patient satisfaction with Neural Therapy (NT) and conventional medicine (COM) in primary care for musculoskeletal diseases.

**Methods:**

A cross-sectional study in primary care for musculoskeletal disorders covering 77 conventional primary care providers and 18 physicians certified in NT with 241 and 164 patients respectively. Patients and physicians documented consultations and patients completed questionnaires at a one-month follow-up. Physicians documented duration and severity of symptoms, diagnosis, and procedures. The main outcomes in the evaluation of patients were: fulfillment of expectations, perceived treatment effects, and patient satisfaction.

**Results:**

The most frequent diagnoses belonged to the group of dorsopathies (39% in COM, 46% in NT). We found significant differences between NT and COM with regard to patient evaluations. NT patients documented better fulfilment of treatment expectations and higher overall treatment satisfaction. More patients in NT reported positive side effects and less frequent negative effects than patients in COM. Also, significant differences between NT and COM patients were seen in the quality of the patient-physician interaction (relation and communication, medical care, information and support, continuity and cooperation, facilities availability, and accessibility), where NT patients showed higher satisfaction. Differences were also found with regard to the physicians' management of disease, with fewer work incapacity attestations issued and longer consultation times in NT.

**Conclusion:**

Our findings show a significantly higher treatment and care-related patient satisfaction with primary care for musculoskeletal diseases provided by physicians practising Neural Therapy.

## Background

Musculoskeletal diseases represent a major health problem throughout the world. No other class of disorders affects more people, leads to a higher prevalence of disability, or places a higher financial burden on health systems [1]. In Switzerland, diseases of the musculoskeletal system and connective tissue account for more than 20 percent of the recorded main diagnoses [2], and as populations age, the prevalence of musculoskeletal diseases is expected to rise rapidly [1,3]. For various reasons, patients with musculoskeletal disorders are increasingly choosing complementary medicine in the search for cures to their problems. The Swiss Federal Department of Home Affairs therefore decided in 1998 to add five methods of complementary medicine, including Neural Therapy (NT), to the benefit catalogue of basic health insurance for a trial period of five years. Reimbursements for expenditures in alternative medicine were covered by basic health insurance only when these methods were provided by physicians with appropriate Complementary and Alternative Medicine (CAM) training approved by the Swiss Medical Association. A nationwide evaluation of several CAM procedures including anthroposophical medicine, homeopathy, traditional Chinese medicine, phytotherapy, and NT was performed to decide about the inclusion of CAM procedures in compulsory health plans beyond the trial period. The project was funded by the Swiss Federal Office of Public Health and a project description and the respective results were published in a final report in 2005 [4]. The goal of the current study was to use patient satisfaction as a measure of the effectiveness of NT in ambulatory care for musculoskeletal diseases as part of the larger project evaluating CAM procedures. The specific research question was: What are the differences between NT and conventional medicine in terms of patient satisfaction and the patient's evaluation of the quality of the patient-physician relationship?

Neural Therapy, according to Huneke, is a treatment that uses precise injections of local anaesthetics for diagnosis and therapy [5,6]. in which pathological stresses (e.g., a vicious circle in pain) are interrupted [7]. This treatment uses the auto-regulatory mechanism of the autonomic nervous system [5-7] mainly on two levels: in a segmental reflectory process and in the so-called interference field, which can cause or maintain pathological processes beyond any segmental order [5,6]. This implies that NT can be divided into a local therapy (e.g., infiltration of trigger points) and a segmental therapy (e.g., therapy of the Head zones, as well as sympathetic ganglia, nerve roots, and peripheral nerves) on the one hand and into an interference field therapy on the other hand. Impulses of the interference field may influence every system of the organism beyond that of the segmental order [5,6,8-11]. The mechanism of the effects of NT is derived from the pathophysiology of pain and from neurophysiological experiments [7,12-17]. Acute and chronic pain and functional abnormalities, all common findings in patients with musculoskeletal disorders, are the main indications for Neural Therapy [5,6].

Within the field of complementary medicine, a considerable amount of research has been done that includes assessments of patient satisfaction [18,19], although little has been done specifically for NT and musculoskeletal disorders. We defined patient satisfaction with the following items: patient-rated symptom relief, fulfillment of treatment expectations, overall treatment satisfaction, frequencies of adverse side effects, and a broad range of aspects of the quality of the patient-physician interaction covered in the EUROPEP questionnaire [20].

## Methods

We examined data related to musculoskeletal diseases as part of a cross-sectional study conducted between 2002 and 2004. In order to provide a picture of routine care, the study was designed to be purely observational, without interference into the treatment choices of patients or the diagnostic/therapeutic procedures of physicians.

### Physicians and patients

**Figure 1:**
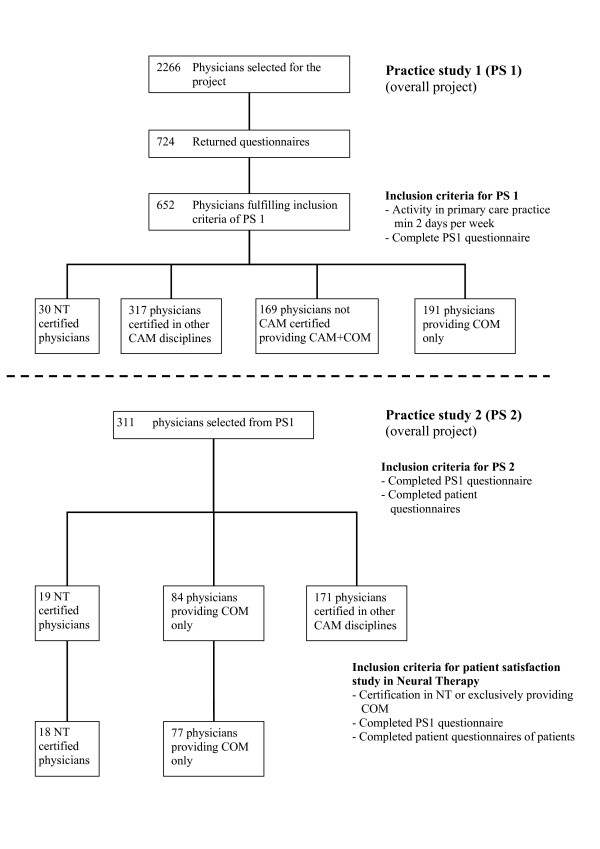
Flowchart of sampling procedures.

A description of sampling procedures used in this study and of how it is embedded in the main project is given in Figure 1. The project consisted of two practice studies. Primary care physicians, including both conventional physicians and those certified by the Swiss Medical Association for NT (SANTH), were invited to participate in the initial study (practice study 1) to evaluate differences in the structure of care. Data regarding professional qualification and certification were also obtained in this phase. The second study (Practice study 2), was aimed at the processes and outcomes of care, and participating physicians were invited to recruit patients [4] (Figure 1). The eligibility criteria for all participating physicians were training and a license in conventional medicine and medical activity in primary care for at least two days per week. A membership list of the SANTH was obtained, and all 41 members with an additional qualification in NT recognized by the Swiss Medical Association (FMH) were asked to participate. These physicians were defined in the study as NT physicians. A random sample taken from the complete list of all Swiss primary care providers, not listed by any medical society for complementary and alternative medicine, was also asked to participate in the project. These physicians were defined as conventional medicine (COM) physicians. The final sample included 18 practitioners who were certificated in NT and 77 COM practitioners.

Physicians and their staff were instructed to sample consecutive patients consulting their practices on four given days during a 12 month period in 2002/03. The sampling days for the individual practices were defined by the study coordination and were equally distributed across seasons and week days. Patients were sampled irrespective of the type of appointment they had with their physicians. Patients were informed about the study by leaflets and prior to a consultation were asked to fill out forms aimed at evaluating demographics, health status, and the frequency and reasons for their health care utilization. Physicians documented the same consultations with reference to symptoms, diagnoses, duration of problems, comorbidities, and diagnostic and therapeutic procedures. The study design ensured that physicians remained unaware of the patient answers.

Outcomes were measured using a questionnaire mailed to patients three to four weeks after the initially documented consultation. The specific outcomes assessed included: symptom relief, fulfilment of treatment expectations, satisfaction with treatment, the presence of adverse and other side effects, and the perceived causality of symptom relief. Participating patients were also given the EUROPEP questionnaire, which was developed by an international group and translated into and validated in multiple languages. This questionnaire has 23 questions each with a 5-point answering scale and covers relation and communication, medical care, information and support, continuity and cooperation, and facilities availability and accessibility. Multiple qualitative and quantitative studies have shown high item response and shown no problems with the translations [21], which is particularly important for Switzerland. All the questionnaires (except the EUROPEP) used in this project were developed in close cooperation with an expert group of Swiss primary care providers specialized in conventional and/or complementary medicine. Questionnaires were sent to patients in their mother tongue (German, French, or Italian) and written informed consent was obtained from all participants. The study was also cleared by the ethics committee of the Canton Bern (Switzerland).

### Data management and data analysis

Only patients over 16 years of age with musculoskeletal disorders (M chapter of ICD-10) were included in the analysis (Figure 1). Data analysis was performed in two steps. The first step included descriptive analyses using tables and graphs and the second step involved the application of analytical procedures. Target variables were analyzed with multivariate linear models. Ordinal outcomes such as the measures of health status, severity and chronicity of disease and all data from the EUROPEP questionnaire were reduced to two-level scales with the most favourable answer category coded as one and all other non-missing categories as zero. These data were analyzed using multivariate logistic regression models. The covariables of the multivariate models were defined a priori and were selected with the aim of adjusting for demographic factors and the durations of patient's health problems. All the analytical procedures accounted for the clustering of observations at the practice level. Taylor series expansion procedures were used for 2*2 tables, and mixed effects models for multivariate procedures [22,23]. These procedures allowed us to account for the variation between physicians in potentially different levels of treatment quality as perceived by patients by assuming that these unobserved physician-specific properties have a random variation. We considered this approach more appropriate than treating specific properties of single physicians as fixed effects. 95% confidence intervals (95% CI) of means, proportions and odds ratios were calculated accordingly.

The level of significance was set at p < 0.05 throughout the study, and the statistical software package SAS 9.1 (SAS Institute Inc., Cary, NC, USA) was used for all calculations.

## Results

### Characteristics of patient population

The participating physicians initially recruited 4093 patients, among whom 786 patients (19.2%) were diagnosed with musculoskeletal disorders and 494 were treated with COM and 292 treated with NT). The proportion of patients with musculoskeletal disorders was 16.1% in COM and 28.5% in NT. A significant difference between COM and NT was observed in the frequency of patients that returned questionnaires (241/494 COM patients (48.8%) and 164/292 (56.2%) NT patients. We enrolled these 405 patients in the study and compiled their complete treatment and outcome information. The proportion of female patients to the whole was 62% in the COM group and 77% in the NT. The average patient age was 55.4 years in the COM group and 53.3 years in the NT (table 1).

**Table 1:** Socio-demography and health status of patient population

		**COM**			**NT**		
		**#**	**%**	**95% CI**^a^	**#**	**%**	**95% CI**^a^
**Patients**	*Number*	241			164		
**Patient age**	*Mean*	55.42			53.28		
**Female Patients**	*Proportion*	150	62.24	55.9–68.6	126	76.83	71–82.7
**Education**	*Proportion of higher education*		20.5	14.9–26.1		24.7	16.4–32.9
**General health**^b^	*excellent*	8	3.45	1.03–5.86	4	2.52	0.00–5.32
	*very good*	33	14.22	10.19–18.26	27	16.98	11.95–22.01
	*good*	126	54.31	47.95–60.67	63	39.62	33.48–45.76
	*fair*	53	22.84	17.75–27.94	59	37.11	27.06–47.15
	*poor*	12	5.17	2.40–7.94	6	3.77	0.00–8.15
**Duration of the main health problem**^b^	*Month (Mean)*		47.7	32.5–63		72.9	48.5–97.2
**Chronic conditions**^b^	*> 3 month*	116	48.1	42.1–54.2	116	70.7	62.8–78.7
**Severe conditions**^b^		77	34.4	29.3–39.5	60	37.7	31.3–44.1

^a ^95% confidence limits

^b^patient rated

Prior to the consultations, patients were asked to rate their general health on a five-point scale ranging from excellent to poor. Answer patterns indicate significant differences in the unadjusted general health status between the COM and NT patient groups (table 1). For statistical analysis, the answer scale was dichotomized into "excellent/very good/good" and "less well/bad". Gender- and age-adjusted differences indicated no significant differences in self-rated general health.

Patients recorded the duration of their main health problem and were classified as acute (duration < 3 months) or chronic (≥ 3 months). The proportion of chronic patients was analyzed using a logistic regression model adjusted for age and gender. This indicated a significantly higher proportion of chronic patients in the NT group (70.7%) than in the COM one (48%).

Patients were further asked to rate the severity of their major health problem using a three-level scale (minor, moderate or serious). A multivariate logistic regression model with patient-reported severity as an outcome (answer level minor coded as 0, and levels moderate and serious coded as 1) indicated no difference in the severity of problems between the NT and COM groups after adjusting for gender, age and the chronicity of disease.

### Prevalence of musculoskeletal disease

The most frequent main diagnoses were categorized into six disease groups (table 2). In both the COM and the NT group, the most frequent diagnoses belonged to the group of dorsopathies (39% in COM, 46% in NT). Table 2 shows the recorded sub-diagnoses in the group of dorsopathies. Using this classification, there were no significant differences between the frequencies of back problems in COM and NT patients.

**Table 2:** Main Diagnoses

**Diseases related to the musculoskeletal system (ICD-10)**		**COM**			**NT**		
		**#**	**%**	**95% CI**^a^	**#**	**%**	**95% CI**^a^
M00–25	Arthropathies	60	24.9	18.7–31.1	30	18.3	10.8–25.8
M30–36	Systemic connective tissue disorders	4	1.66	0.1–3.2	3	1.8	0.2–3.4
M40–54	Dorsopathies	95	39.4	32.5–46.3	76	46.3	35.1–57.6
M60–79	Soft tissue disorders	72	29.9	23.3–36.4	49	29.9	20.2–39.5
M80–94	Osteopathies and chondropathies	5	2.07	0.4–3.8	2	1.2	0.0–3.0
M95–99	Other disorders of the musculoskeletal system and connective tissue	5	2.07	0.4–3.8	4	2.4	0.0–5.4

**Back-pain diagnoses (M40–M54)**		95	39.4	32.5–46.3	88	53.7	35.1–57.6

M43	Other deforming dorsopathies	4	1.7		1	0.6	
M45	Ankylosing spondylitis	1	0.4		0	0	
M47	Spondylosis	3	1.2		3	1.8	
M48	Other spondylopathies	3	1.2		1	0.6	
M51	Other intervertebral disc disorders	6	2.5		4	2.4	
M53	Other dorsopathies, not elsewhere classified	3	1.2		15	9.2	
M54	Dorsalgia	75	31.1		52	31.7	

Comorbid conditions							

*None*		101	41.9	34.3–49.6	51	31.1	20.8–41.4
*1*		61	25.3	19.8–30.8	58	35.4	29.4–41.3
*> 1*		79	32.8	25–40.6	55	33.5	21.7–45.4

^a ^95% confidence limits

Dorsopathies were followed by soft tissue disorders (30% in both groups), arthropathies (25% in COM, 19% in NT), osteopathies and chondropathies, other disorders of the musculoskeletal system and connective tissue, and systemic connective tissue disorders. The most frequently recorded ICD-10M diagnoses for COM were radiculopathy, low back pain, and cervicalgia. The most frequent diagnoses for NT were cervicalgia, myalgia (not elsewhere classified), and cervicobrachial syndrome.

The concomitance of comorbidities allows for a measure of disease severity. The distribution of the frequency of comorbidities is shown in table 2. There was no statistically significant difference between COM and NT.

### Patient attitudes and expectations (table 3)

**Table 3:** Patient expectations

		**COM**			**NT**		
		**#**	**%**	**95% CI**^a^	**#**	**%**	**95% CI**^a^
**Expectations**	*Healing*	112	46.5	40.1–53.8	82	50.0	41.3–58.7.0
	*Alleviation*	128	53.1	47.5–58.8	86	52.4	42.4–62.5
	*Agreeable method*	5	2.1	0.3–3.8	2	1.2	0.0–3.5
	*Less adverse side effects*	22	9.1	5.1–13.1	30	18.3	11.2–25.4
	*Lower costs*	2	0.8	0.0–2.0	4	2.4	0.3–4.5

^a ^95% confidence limits

No differences were found between the COM and NT groups with respect to the patient's expectations for healing, symptom relief, agreeable method, side effects and lower cost.

### Management of disease (physicians)

No significant differences between groups were observed for diagnostic and therapeutic referrals to other physicians and specialists (10.5% in NT, 13.3% in COM). However, NT physicians had significantly longer consultations than their COM peers (19.1 minutes in NT, 17.1 minutes in COM). Our results also show that NT physicians issued work incapacity attestations for a population of patients under 65 less often than COM physicians (3.2% in NT, 17.0% in COM).

### Patient evaluations (table 4)

**Table 4:** Patient evaluations

		**COM**			**NT**		
		**#**	**%**	**95% CI**^a^	**#**	**%**	**95% CI**^a^
**Symptom relief**	Complete resolution*	30	12.9	7.8–18.1	12	7.4	3.1–11.7
	*Considerably weaker*	68	29.3	23.5–35.2	64	39.5	32.9–46.1
	*Somehow weaker*	63	27.2	20.8–33.5	47	29.0	22.0–36.0
	*Unchanged*	61	26.3	20.7–31.9	34	21	16.2–25.8
	*Very intense*	9	3.9	1.4–6.4	4	2.5	0.2–4.7
	*Unsupportable*	1	0.4	0–1.3	1	0.6	0–1.8
Fulfillment of treatment expectations*	*Proportion of "complete fulfilled"*	33	15	9.6–20.5	40	24.8	18.5–31.2
Overall Satisfaction*	*Proportion of "very satisfied"*	66	29.5	22.7–36.2	66	41.3	35.9–46.6
**Adverse side effects**	*Yes*	29	12.9	8.4–17.5	17	10.6	8.2–12.9
**Severity of side effects**	*weak*	4	13.8^b^	1.6–26.0	7	41.2	13.1–69.2
	*moderate*	18	62.1	45.2–78.9	5	29.4	6.9–52.0
	*strong*	7	24.1	7.6–40.7	5	29.4	7.7–51,2
Other effects*	*Positive*	38	17.7	13.1–22.3	59	37.6	29.1–46.1
	*Negative*	16	7.4	3.8–11.1	9	5.7	2.5–9.0
	*No effect*	161	74.9	70.3–79.5	89	56.7	47.0–66.4
Perceived causality of symptom relief*	*Very sure*	59	29.7	22.2–37.1	62	44.3	36.0–52.6
	*Sure*	77	38.7	31.0–46.4	55	39.3	29.9–48.7
	*Not sure*	39	19.6	13.7–25.5	12	8.6	4.1–13.1
	*Don't know*	24	12.1	7.0–17.1	11	7.9	3.1–12.6

*significant difference (p < 0.05) in the proportion of the most favorable answer option between NT and COM in a multivariate logistic model (age, gender and chronicity controlled)

^a ^95% confidence limits

^b ^percentage of patients with adverse side effects within group

Unadjusted results for symptom relief indicated generally poorer outcomes for NT patients. However, after adjusting the analysis for gender, age, and the chronicity of disease, no significant differences were observed for the proportion of patients with a complete resolution of symptoms.

The fulfillment of treatment expectations was rated using a four-point scale ranging from complete fulfillment to not at all. Unadjusted comparison of frequency indicated no significant difference in the fulfillment of expectations between the groups. However, after analysis with an age-, gender-, and chronicity-adjusted logistic regression model of complete fulfillment of expectations, a significantly better outcome was observed for NT patients.

The unadjusted general treatment satisfaction, rated from very satisfied to not satisfied at all showed significant differences between groups. The age-, gender-, and chronicity-adjusted frequencies of very satisfied answers indicated significantly higher treatment satisfaction in NT patients.

The majority of COM (87%) and NT (89.4%) patients did not report treatment-related adverse side effects, and the difference between groups was not significant. Differences between groups were seen in the severity of side effects with a tendency for less severe effects in the NT group (table 4). NT patients also reported additional positive effects not directly related to their main health problems, and observed other additional effects with less frequency.

The causality of symptom relief was analysed by asking patients to what degree they considered symptom amelioration to be the consequence of a specific treatment. There were significant differences between the groups with NT patients more often confident that the amelioration of their symptoms was as a result of the treatment performed (table 4).

### EUROPEP questionnaire (table 5)

**Table 5:** Patient satisfaction (EUROPEP)

**Questions/items**	**COM**		**NT**	
	
	**%**	**95% CI**^a^	**%**	**95% CI**^a^
**Relation and communication**				
1. *Making you feel you had time during consultation?*	60.6	53.9–67.3	70.3	53.3–87
2. Interest in your personal situation?*	58.6	52.1–65	72	60–83.9
3. Making it easy for you to tell him or her about your problem?*	61.1	53.2–69	71.9	59–84.8
4. *Involving you in decisions about your medical care?*	55.7	48.1–63.4	64.7	54.2–75.1
5. *Listening to you?*	65.8	59.1–72.5	73.7	61.1–86.3
6. Keeping your records and data confidential?*	69.5	62.4–76.7	83.1	74.6–91.6

**Medical care**				
7. Quick relief of your symptoms?*	16.1	10.3–22	26.6	20.3–32.9
8. Helping you to feel well so that you can perform your normal daily activities?*	27	20.2–33.8	40.7	32.7–48.8
9. Thoroughness?*	50.5	43.1–57.8	68.4	59.6–77.1
10. *Physical examination of you?*	47.1	40.7–53.4	55.7	38.8–72.6
11. *Offering you services for preventing diseases (screening, health checks, immunizations)?*	43	34.2–51.9	54.3	41.9–66.7

**Information and support**				
12. Explaining the purpose of tests and treatments?*	57.1	50.2–64.1	72.7	64.9–80.5
13. Telling you what you wanted to know about your symptoms and/or illness?*	55.4	48.1–62.7	71.2	60.4–81.9
14. *Helping you deal with emotional problems related to your health status?*	47.8	40.3–55.2	57.9	44.6–71.2
15. Helping you understand the importance of following his or her advice?*	44.1	37.2–51.1	61.7	51.4–71.9

**Continuity and cooperation**				
16. *Knowing what s/he had done or told you during earlier contacts?*	53.2	45.2–61.2	63.5	50.3–76.7
17. Preparing you for what to expect from specialist or hospital care?*	53.4	44.9–61.8	76.8	64.9–88.6

**Facilities availability and accessibility**				
18. The helpfulness of the staff (other than the doctor)?*	64.3	57.9–70.7	74.8	65.8–83.9
19. *Getting an appointment to suit you?*	1.3	0–2.7	1.2	0.0–3.1
20. *Getting through to the practice on telephone?*	73.5	65.9–81.1	69.1	56.5–81.8
21. *Being able to speak to the general practitioner on the telephone?*	54.9	46.4–63.3	58.9	42.3–75.6
22. *Waiting time in the waiting room?*	36.3	27.8–44.8	42.8	31.4–54.2
23. *Providing quick services for urgent health problems?*	67.7	59.7–75.6	70.2	61.5–79.0

*significant difference in the proportion of the most favorable answer option (p < 0.05) between NT and COM in a multivariate logistic model (age, gender and chronicity controlled)

^a ^95% confidence limits

For all EUROPEP questions, with the exception of 10, 19, and 20, NT patients indicated higher satisfaction and the differences between the groups for questions 2, 3, 6, 7, 8, 9, 12, 13, 15, 17, and 18 were statistically significant.

## Discussion

This study was part of a nationwide evaluation of complementary medicine in Swiss primary care. Its specific objective was to assess patient satisfaction for the treatment of musculoskeletal diseases with Neural Therapy compared to conventional medicine. Although NT accounts for only a small fraction of Swiss primary care; in 2002 only 1.2% of all primary care providers (69 practitioners) had the respective certification, our results may nevertheless provide some important information for the treatment of musculoskeletal diseases, and, in particular, spine problems. The considerable difference we observed between COM and NT in the proportion of patients with musculoskeletal problems indicates a certain attraction to NT that other research confirms [5,6,24-28]. Our study may be criticized for data that are mainly based on perceived health status and self-reported, subjective assessments of patients, but such evaluations have proven to be valid measures of health in general populations [29,30]. A related, important finding is that NT patients considered their general health and the severity of major health problems to be the same as those of COM patients, whereas other findings on complementary medicine report poorer general health and more severe disease conditions for NT patients compared to COM patients [8]. However, in accordance with the literature NT patients more often reported chronic health problems than COM patients [31-34].

The management of musculoskeletal disorders appeared to be different in NT and conventional primary care. NT physicians had longer consultations, but they issued work incapacity attestations less often than their conventional colleagues. These have consequences on the direct and indirect costs of treatment. Longer consultations, which are likely the result of the invasive character of NT, increase the direct costs, but fewer work incapacity attestations may result in lower indirect costs. Musculoskeletal disorders, especially those related to back-pain, cause an enormous economic burden on health care systems [1], and back-pain itself appears to be a major factor in lost work productivity [35]. Our data therefore provide some empirical evidence that increased use of NT in primary care may result in lower health care expenditures in Swiss ambulatory care.

Our interpretation of patient satisfaction data discriminated between treatment-related outcomes (symptom relief, fulfillment of treatment expectations, adverse side effects, and other effects) and care-related outcomes (overall satisfaction with treatment, and the EUROPEP questionnaire) is that there are equal or better outcomes when measured by treatment-related items, and also consistently better outcomes for all care-related items. However, some contradictions were seen in the patients' evaluations of symptom relief. No difference was found between the groups when patients were asked about symptom relief, but significantly more NT patients reported quick relief of symptoms (EUROPEP-question 7).

We did not find a difference between NT and COM with respect to patient based reports of adverse side effects. The observations that patients in NT have more often reported positive effects and COM patients have more often perceived negative effects may reinforce the idea that patient expectations and outcomes are linked [36,37]. However, it has to be acknowledged that the reports of adverse side effects were based on evaluations by patients and not on more objective clinical criteria.

It can be argued that the consistently higher satisfaction expressed in the EUROPEP questionnaire by NT patients is likely the result of longer consultation times in NT [38], although there is some debate about which aspects of the physicians behaviour affect patient-based evaluations in primary care [39]. However, some particular characteristics of NT need to be considered in this context. The first part of a consultation in NT, consisting of anamnesis and examination, is comparable to conventional medicine and requires the same amount of time. The second part of a NT consultation usually includes an injection that actually harms the patient. Longer consultations in NT are therefore not related to extended communication time between patients and physicians but are mostly due to the duration of a specific therapeutic procedure.

### Limitations of the study

Several limitations of the study can be acknowledged. With reference to external validity, the extent to which the study results can be generalized is limited by the small sample size of patients with musculoskeletal diseases (241-COM, 164-NT) and their physicians (77-COM, 18-NT). It is impossible to asses the validity of this sample size as the overall distribution of patients with musculoskeletal problems in Swiss primary care is unknown.

However, other research within the larger project showed that the original samples of physicians and patients, irrespective of diagnosis related inclusion criteria, were reasonably representative of their respective base populations [2,40].

Potential sources of bias in this study include the fact that satisfied and dissatisfied patients have different compliance in completing questionnaires. It is therefore very likely that the results are positively biased because satisfied patients are more likely to return the questionnaires [41,42].

The study was based on a cross-sectional design and patients received a questionnaire 3–4 weeks after a single consultation, irrespective whether they had other appointments with a physician within or outside the study. This implies that the description and comparison of the satisfaction of the two patient groups may not fully account for effectiveness of procedures in an ongoing therapeutic process. It may also be possible that work incapacity attestations were either pre-existing or issued by other, non-study physicians.

Other limitations of the study are related to the fact that outcome measures such as the EUROPEP are not specific for musculoskeletal disorders and may not be appropriate for assessing patient satisfaction with NT. The broad range of outcomes assessed in the survey, which was not originally intended to specifically evaluate diseases treated by NT, made it impossible to investigate essential issues as deeply as would have been possible in a survey designed specifically to assess patient satisfaction with NT. An evaluation of long-term effects and chronic disease, which is also important, was not in the scope of the overall project.

Another problem of the study relates to the limitations in diagnosing musculoskeletal diseases in a primary care setting where sophisticated imaging is usually not available. Such limitations are, however, present in both COM and NT. Bias as a result of uneven diagnostic certainty across study groups may therefore not be a concern.

Patient questions other than the EUROPEP questionnaire were not validated. Limited temporal and financial resources in the project made a respective psychometric validation for three different languages prohibitive. However, literature provides support that patient evaluations of care give valid estimates of their experiences and respective satisfaction in a primary care setting [43,44]. It may also be criticized that outcomes were dichotomized into the best possible and all other answer options. This approach is based on a commonly applied concept that standards of excellence attained by top performers should be used as benchmarks of quality in the health care sector [45].

Finally, it can be argued that the analysis of our outcome data needs adjustment for the problem of multiple tests. The literature in this field is inconclusive [46] and the decision whether to view the EUROPEP data as a group or as individual questions remains arbitrary. This study has the character of a pilot study and we therefore promote a more informal use of the hypothesis tests, which implies that the results are important but the p-values have little meaning. Consequently, it is possible that our results accidentally mislead the interpretation of individual questions as significant p-values can occur by chance alone. However, the EUROPEP results showed consistently better outcomes for NT in almost all questions and we consider it unlikely that the overall interpretation of the data was affected by accidentally significant p-values.

## Conclusion

In light of the high prevalence of musculoskeletal disorders, our results that show significantly higher patient satisfaction in NT may have practical importance. Better patient satisfaction and better fulfillment of expectations along with an equal patient-perceived efficacy in the treatment of musculoskeletal disorders make NT treatment an option for primary care or referring physicians. Increased application of neuraltherapeutics by primary care physicians could therefore be considered reasonable and increased integration of NT in medical education would also be justified. However, further research is needed to establish outcomes that are more evidence based, and to provide a more in-depth analysis of the cost-effectiveness of NT.

## Competing interests

The Swiss Federal Office of Public Health funded the project and by contract researchers were independent from the funding agency. The authors declare that they have no competing interests.

## Authors' contributions

JM wrote the first draft of the manuscript. LF and LS reviewed and completed the manuscript and provided considerable input with reference to neuraltheapy and musculoskeletal health problems. AB obtained the mandate of the project, performed all statistical analyses and completed the manuscript in this context. All authors read and approved the final manuscript.

## Pre-publication history

The pre-publication history for this paper can be accessed here:



## References

[B1] Smolen J (2004). Combating the burden of musculoskeletal conditions. Ann Rheum Dis.

[B2] Busato A, Donges A, Herren S, Widmer M, Marian F (2005). Health status and health care utilisation of patients in complementary and conventional primary care in Switzerland–an observational study. Fam Pract.

[B3] WHO (2003). The burden of musculoskeletal conditions at the start of the new millennium. World Health Organ Tech Rep Ser.

[B4] Melchart D, Mitscherlich F, Amiet M, Eichenberger R, Koch P (2005). Programm Evaluation Komplementärmedizin (PEK) Schlussbericht. Health SFOoP.

[B5] Barop H (1996). Lehrbuch und Atlas der Neuraltherapie nach Huneke.

[B6] Fischer L (2001). Neuraltherapie nach Huneke. Grundlagen, Technik, praktische Anwendung.

[B7] Fischer L (2003). Pathophysiologie des Schmerzes und Neuraltherapie. PRAXIS.

[B8] Breebart A, Bijlsma J, Van Eden W (2002). 16-year remission of rheumatoid arthritis after unusually vigorous treatment of closed dental foci. Clin Exp Rheumatol.

[B9] Iida M, Yamaguchi J (1985). Remission of rheumatoid arthritis following periodontal treatment. A case report. Nippon Shishubio Gakkai Kaisha.

[B10] Kea P (1993). Missing teeth and ischemic heart disease in men aged 45–64 years. Eur Heart J.

[B11] Pohle S (1992). Odontogene Störfelder als Ursache für periphere Erkrankungen – Eine neuraltherapeutische Studie. Ärztez f Naturheilverf.

[B12] Baron R, Jänig W (1998). Schmerzsyndrome mit kausaler Beteiligung des Sympathikus. Anästhesist.

[B13] Melzack R, Wall P (1965). Pain-mechanisms. A new theory. Science.

[B14] Schattschneider J, Wasner G, Binder A, Siebrecht D, Baron R (2003). Das Symptom "sympathisch unterhaltener Schmerz". Schmerz.

[B15] Zieglgänsberger W (2002). Chronischer Schmerz: Physiologie, Pathophysiologie und Pharmakologie. Ganzheitsmed.

[B16] Ricker G (1924). Pathologie als Naturwissenschaft – Relationspathologie.

[B17] Speranski A (1950). Grundlage einer Theorie der Medizin. Ins Deutsche übertragen von Roques KR.

[B18] Furnham A, Forey J (1994). The attitudes, behaviors and beliefs of patients of conventional vs. complementary (alternative) medicine. J Clin Psychol.

[B19] Kaptchuk T, Eisenberg D (2001). Varieties of healing. 1: medical pluralism in the United States. Ann Intern Med.

[B20] Grol R, Wensing M, Mainz J, Ferreira P, Hearnshaw H, Hjortdahl P, Olesen F, Ribacke M, Spenser T, Szecsenyi J (1999). Patients' priorities with respect to general practice care: an international comparison. European Task Force on Patient Evaluations of General Practice (EUROPEP). Fam Pract.

[B21] Grol R, Wensing M (1999). Task force on patient evaluations of general practice care.

[B22] Singer J (1998). Using SAS PROC MIXED to fit multilevel models, hierarchical models, and individual growth models. Journal of Educational and Behavioral Statistics.

[B23] Sheu C (2002). Fitting mixed-effects models for repeated ordinal outcomes with the NLMIXED procedure. Behav Res Methods Instrum Comput.

[B24] Barbagli P, Bollettin R (1998). [Therapy of articular and periarticular pain with local anesthetics (neural therapy of Huneke). Long and short term results]. Minerva Anestesiol.

[B25] Becke H (1995). Neuraltherapie und Kreuzschmerzen. Überlegungen zur Ursache und Ergebnisse einer Behandlungsstudie. Natura-med.

[B26] Haaks TTW (1999). Neuraltherapeutische Behandlung der schmerzhaften hemiparetischen Schulter. Bologische Medizin.

[B27] Von Orelli F (1999). Die Behandlung chronischer Schmerzen mit Procaininjektionen. Der informierte Arzt/Gazette Medical.

[B28] Eder M (1983). [Pathology. Central problem: biologic follow-up]. MMW Munch Med Wochenschr.

[B29] Miilunpalo S, Vuori I, Oja P, Pasanen M, Urponen H (1997). Self-rated health status as a health measure: the predictive value of self-reported health status on the use of physician services and on mortality in the working-age population. J Clin Epidemiol.

[B30] Hunt SMMKS, Mc Ewen J, Williams J, Papp E (1981). The Nottingham Health Profile: subjective health status and medical consultations.

[B31] Astin JA (1998). Why patients use alternative medicine: results of a national study. Jama.

[B32] Kersnik J, Svab I, Vegnuti M (2001). Frequent attenders in general practice: quality of life, patient satisfaction, use of medical services and GP characteristics. Scand J Prim Health Care.

[B33] Shmueli A, Shuval J (2004). Use of complementary and alternative medicine in Israel: 2000 vs. 1993. Isr Med Assoc J.

[B34] Bürgi M, Sommer J, T R (1996). Alternative Heilmethoden: Verbreitungsmuster in der Schweiz.

[B35] Schmidt C, Kohlmann T (2005). [What do we know about the symptoms of back pain? Epidemiological results on prevalence, incidence, progression and risk factors]. Z Orthop Ihre Grenzgeb.

[B36] Mondloch M, Cole D, Frank J (2001). Does how you do depend on how you think you'll do? A systematic review of the evidence for a relation between patients' recovery expectations and health outcomes. Cmaj.

[B37] Crow R, Gage H, Hampson S, Hart J, Kimber A, Thomas H (1999). The role of expectancies in the placebo effect and their use in the delivery of health care: a systematic review. Health Technol Assess.

[B38] Freeman GK, Horder JP, Howie JG, Hungin AP, Hill AP, Shah NC, Wilson A (2002). Evolving general practice consultation in Britain: issues of length and context. Bmj.

[B39] Thorne SE, Harris SR, Mahoney K, Con A, McGuinness L (2004). The context of health care communication in chronic illness. Patient Educ Couns.

[B40] Busato A, Eichenberger R, Kuenzi B (2006). Extent and structure of health insurance expenditures for complementary and alternative medicine in Swiss primary care. BMC Health Serv Res.

[B41] Ferris L (1992). A guide to direct measures of patient satisfaction in clinical practice. Health Services Research Group. Cmaj.

[B42] Mazor KM, Clauser BE, Field T, Yood RA, Gurwitz JH (2002). A demonstration of the impact of response bias on the results of patient satisfaction surveys. Health Serv Res.

[B43] Turnbull JE, Hembree WE (1996). Consumer information, patient satisfaction surveys, and public reports. Am J Med Qual.

[B44] Wensing M, Vedsted P, Kersnik J, Peersman W, Klingenberg A, Hearnshaw H, Hjortdahl P, Paulus D, Kunzi B, Mendive J (2002). Patient satisfaction with availability of general practice: an international comparison. Int J Qual Health Care.

[B45] Weissman NW, Allison JJ, Kiefe CI, Farmer RM, Weaver MT, Williams OD, Child IG, Pemberton JH, Brown KC, Baker CS (1999). Achievable benchmarks of care: the ABCs of benchmarking. J Eval Clin Pract.

[B46] Thompson JR (1998). Invited commentary: Re: "Multiple comparisons and related issues in the interpretation of epidemiologic data". Am J Epidemiol.

